# Calcifying odontogenic cyst associated with the impacted third molar: a case report

**DOI:** 10.11604/pamj.2019.33.151.17601

**Published:** 2019-06-27

**Authors:** Tuğçenur Uzun, Ertunç Çinpolat

**Affiliations:** 1 Abant Izzet Baysal University, Faculty of Dentistry, Department of Oral and Maxillofacial Surgery, Bolu, Turkey; 2 Abant Izzet Baysal University, Faculty of Medicine, Department of Pathology, Bolu, Turkey

**Keywords:** Calcifying odontogenic cyst, impacted third molar, cone beam computed tomography

## Abstract

Calcifying odontogenic cyst (COC) is a benign, locally aggressive, slow-growing lesion. Its occurrence constitutes about 2% of all odontogenic cysts. The most frequent sign is painless, slow growing swelling. Radiographically it appears as a well-defined unilocular radiolucency. The microscopical features of lesion showed well-delineated cystic proliferation of odontogenic epithelium with ghost cells and fibrous connective tissue wall. In the present study, 42 years old man who has COC associated with the impacted third molar treated with enucleation is reported after 6 months follow-up.

## Introduction

Calcifying odontogenic cyst (COC) was defined by Pindborg in 1962 as an analog to calcifying epithelioma in the skin [1]. In 1971, World Health Organization (WHO) defined the lesion as a non-neoplastic cystic lesion and named it as COC. In 1992, WHO classified the lesion as odontogenic tumor, but did not change its name. In 2005, it was renamed as calcifying cystic odontogenic tumor (CCOT). In the new 4^th^ edition of WHO classification in 2017, the consensus group classifies the cyst as calcifying odontogenic cyst and the neoplasm as dentinogenic ghost cell tumour. COC is a developmental cyst with odontogenic origin, and constitutes 2% of all odontogenic tumors [2]. Clinically, it is characterized by slowly growing asymptomatic swelling. It may have a central (intraosseous), or less frequently, a peripheral (extraosseous) localization [3]. It may cause lingual expansion, displacement of teeth, root resorption, and perforation in the cortical bone [4]. Radiographically, it may have unicystic or multicystic well-bordered radiolucent appearance. It may contain irregular radiopaque foci. Nearly half of the cases are associated with an unerupted tooth [5,6]. It affects maxilla and mandible in a similar rate, and there is not a difference between sexes or races [7,8]. It often affects the anterior of the first molar teeth [9]. It can be observed in a wide age range, but it is more frequent in the second decade. The youngest reported case was 2 days old [10]. It is difficult to diagnose COC with clinical and radiographic features alone. The diagnosis is only possible with histological examination.

## Patient and observation

**Figure 1:**
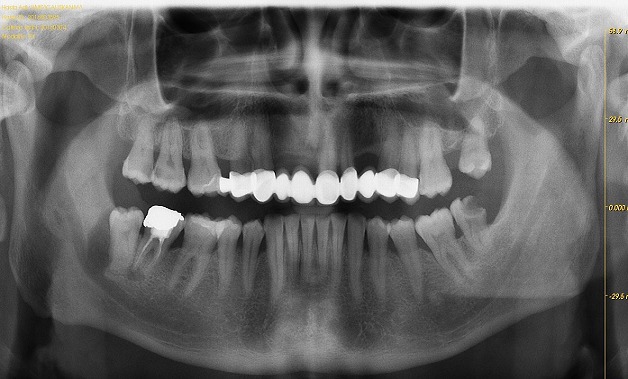
Preoperative panoramic radiograph of the lesion

**Figure 2:**
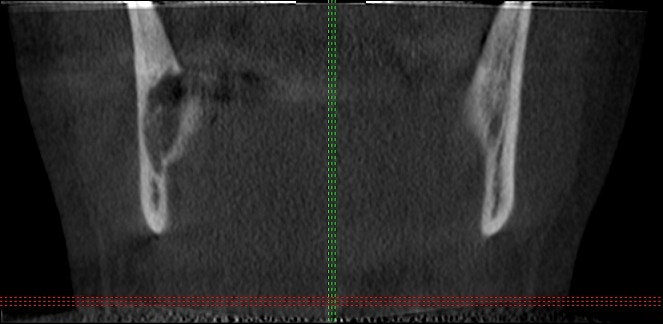
Cone beam computed tomography (CBCT) image of the lesion

**Figure 3:**
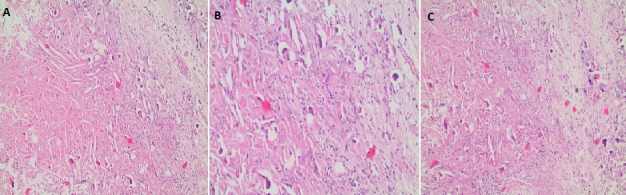
Histologic view of the lesion (A, B, C)

**Figure 4:**
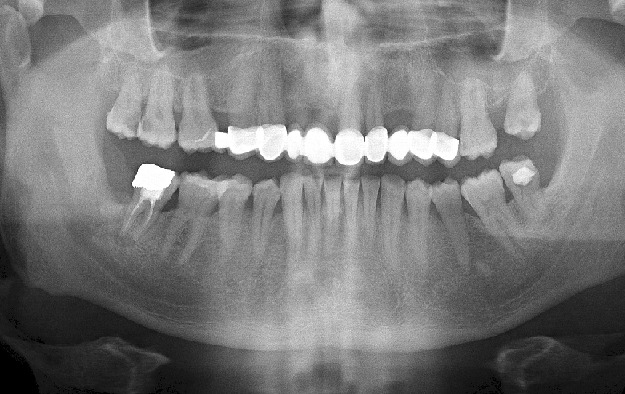
Six months postoperative radiograph

A 42 years old, systemically healthy male visited the Department of Oral and Maxillofacial Surgery, School of Dentistry, University of Abant Izzet Baysal, Bolu, Turkey for a routine check-up. His panoramic radiogram revealed an irregularly bordered radiolucent lesion with approximately 2cm diameter around the impacted right mandibular wisdom tooth (Figure 1). A cone beam computed tomography (CBCT) was performed to examine the lesion in detail. CBCT images showed calcification foci and close relationship of the lesion with inferior alveolar nerve (Figure 2). There was no sign of a lesion in intraoral and extraoral inspection. The mucosa around the lesion area had normal color, and bone hardness was felt at the area. The patient did not have any symptoms such as pain or paresthesia. The lesion was enucleated under local anesthesia and was sent to histopathological examination. Histopathological examination of the sections revealed dense, mixed inflammatory cell infiltration and diffuse psammomatous calcification in the fibrous tissue specimens that had walled structure. There were ghost cells in one focus (Figure 3). At the end of 6 months, the lesion healed without problems, and the patient is currently under follow-up (Figure 4).

## Discussion

From the time it has been described, COC has led to many controversies regarding its nature, terminology and classification. The main reason for these controversies is the presence of two different forms as cystic and neoplastic lesions. Pretorius *et al.* classified the lesion as cyst and neoplasm and proposed the term dentinogenic ghost cell tumor for the neoplastic variant [7]. The prevalence of the cystic form is 86%-98%, whereas the neoplastic form is less common and constitutes 2%-16% of cases [11]. There is no specific sign of the lesion either clinically or radiographically. Differential diagnoses include dentigerous cyst, central giant cell granuloma, keratocystic odontogenic tumor and ameloblastoma, which have benign radiolucent appearance. In 20% of cases, COC is associated with odontoma and ameloblastoma [12]. Histologically, they are defined as cystic proliferations with fibrous connective tissue surrounded by odontogenic epithelium. The characteristic sign for COC is the presence of ghost cells and calcifications. Ghost cells have nuclear remnants. Calcifying odontogenic cyst is a pathology that may be associated with impacted teeth [13]. Mortazavi *et al.* reported that the least common pathology associated with impacted teeth is calcifying odontogenic cyst [14]. It has also been reported that calcifying odontogenic cyst is often associated with anterior impacted teeth. The treatment of COC is simple enucleation and curettage. Long-term follow-up with radiograms is recommended for solid neoplastic forms. There have been reports of cases that developed recurrence after 8 years from enucleation. Cases with a long history and those that develop recurrence carry risk for malignant transformation. Li *et al.* reported a case of COC with recurrence that showed transformation to giant cell odontogenic carcinoma [15]. This points out to the importance of follow-up.

## Conclusion

Oral and maxillofacial surgeons should be careful about the radiolucencies associated with impacted third molars.

## Competing interests

The authors declare no competing interests.
